# Diagnostic Agreement in Subepidermal Blistering Diseases: Is ELISA Test Reliable as Direct Immunofluorescence? A Systematic Review, Meta‐Analysis, and Trial Sequential Analysis

**DOI:** 10.1111/jop.70088

**Published:** 2025-12-12

**Authors:** Romeo Patini, Gioele Gioco, Michele Davide Mignogna, Stefania Leuci, Francesco Scilla, Davide Gentile, Cosimo Rupe, Enrico Russo, Anna Schiavelli, Carlo Lajolo

**Affiliations:** ^1^ Head and Neck Department, Fondazione Policlinico Universitario A. Gemelli—IRCCS, School of Dentistry Università Cattolica del Sacro Cuore Rome Italy; ^2^ Department of Neuroscience, Reproductive and Odontostomatological Sciences, Oral Medicine Unit University of Naples Federico II Naples Italy; ^3^ Oral Surgery Postgraduate School, Department of Medical Biotechnologies University of Siena Siena Italy; ^4^ Department of Chemical Science and Technologies, PhD in Materials for Sustainable Development – Dentistry University of Rome Tor Vergata Rome Italy; ^5^ Research Unit in Periodontology and Periodontal Medicine – Department of Clinical and Experimental Medicine University of Florence Florence Italy

## Abstract

**Objectives:**

Direct immunofluorescence (DIF) is the gold standard for diagnosing subepidermal blistering diseases (SBDs). However, DIF requires specialized expertise; therefore, alternative immunological methods such as enzyme‐linked immunosorbent assays (ELISA) are worth exploring. The aim of this review was to evaluate the diagnostic agreement between DIF and ELISA for the diagnosis of SBD.

**Materials and Methods:**

A systematic literature review of international journals and electronic databases (MEDLINE via OVID, Scopus, and Web of Science) was conducted between their inception and December 2023. The risk of bias and overall quality of evidence were assessed using the Newcastle‐Ottawa Scale and the GRADE system.

**Results:**

Of the 1691 articles identified, 38 were included in the qualitative synthesis and 37 in the quantitative analysis. Three meta‐analyses were developed and revealed the superiority of DIF over ELISA, with the following risk differences: BP180, 0.26 (95% CI: 0.19–0.33, *p* < 0.00001, *I* ^2^ : 96%); BP230, 0.59 (95% CI: 0.47–0.71, *p* < 0.00001, *I* ^2^ : 98%); and Lam332, 0.82 (95% CI: 0.70–0.94, *p* < 0.00001, *I* ^2^ : 89%).

**Conclusions:**

DIF remains the gold standard for the diagnosis of autoimmune SBDs. ELISA can serve as a complementary diagnostic tool, especially as a follow‐up instrument for patients with SBD, owing to its low invasiveness.

## 1 Introduction

Autoimmune blistering diseases (ABDs) are a heterogeneous group of illnesses caused by autoantibodies against epithelial cell–cell and cell–matrix adhesion proteins, resulting in chronic skin lesions, mainly bullae and/or oral ulcers; oral blisters are rarely detected, because they soon rupture and form ulcers [1]. Circulating antibodies interact with antigens and trigger an intricate inflammatory reaction that leads to the detachment of cell–cell or cell‐basal membrane contacts in the skin and/or mucous membranes from the underlying collagen, resulting in skin bullae or oral ulcers; other evidence suggests that complex intracellular signaling pathways contribute to the detachment of cell–cell or cell‐connective tissue adhesions [2].

ABDs are classified into two groups, intraepithelial blistering diseases (IBDs) and subepidermal blistering diseases (SBDs), as shown in Table 1 [2, 3, 4, 5]. In IBDs, the loss of adhesion between keratinocytes occurs, with intraepidermal split formation, whereas in SBDs, autoantibodies are attached along the dermal–epidermal junction, causing subepidermal blistering. The antigens involved in these diseases are shown in Table 1.

**TABLE 1:** Summary of ABDs and related autoantigens.

Autoimmune blistering disease	Autoantigens	DIF	ELISA
Intraepithelial blistering diseases			
Pemphigus vulgaris	Dsg1 and Dsg3	A typical net‐like intercellular substance pattern with anti‐IgG and anti‐C3	Titer of IgG anti‐ Dsg1 and Dsg3
Pemphigus foliaceus	Dsg1 and Dsg3	As pemphigus vulgaris	Titer of IgG anti‐ Dsg1 and Dsg3
IgA pemphigus	IgA against keratinocytes	A typical net‐like intercellular substance pattern with anti‐IgG and anti‐C3, and rarely also with anti‐IgA	IgA against keratinocytes
Paraneoplastic pemphigus	Evoplakin, periplakin, plectin, Dsg1, Dsg3, desmocollin 1 and 3, alpha‐2‐macroglobulin‐like‐1, BP180, BP230	As pemphigus vulgaris	Titer of IgG anti‐ Dsg1 and Dsg3
Subepithelial blistering diseases			
Bullous pemphigoid	BP180 and BP230	Linear deposition of immunoglobulins (IgG, IgA) or complement (C3) along the basement membrane zone (BMZ) of epithelial tissues	BP ELISA with NC16A‐BP180 or BP230 recombinant antigen
Pemphigoid gestationis	BP180 and BP230	linear deposition of immunoglobulins (IgG, IgA) or complement (C3) along the basement membrane zone (BMZ) of epithelial tissues	BP ELISA with NC16A‐BP180 or BP230 recombinant antigen
Mucous membrane pemphigoid	Laminin 332, α6β4 integrin and BP180 annd BP230	Linear deposition of immunoglobulins (IgG, IgA) or complement (C3) along the basement membrane zone (BMZ) of epithelial tissues	ELISA tests are designed to detect antibodies against specific BMZ components, including BP180, laminin 332
Anti‐p200/laminin‐γ1 pemphigoid	Laminin γ1	Linear IgG and C3 deposits along the dermal‐epidermal junction in both bullous pemphigoid and anti‐laminin γ1 pemphigoid	The C‐terminus of laminin‐γ1 is the immunodominant region of the protein
Linear IgA disease	IgA anti‐basement against the 97 kDa portion of BPAG2 and 120 kDa LAD‐1	Linear deposit of IgA	IgA reactivity against the BP180 ectodomain
Epidermolysis bullosa acquisita	Collagen VII	Linear immune deposit pattern along the cutaneous BMZ. IgG and C3 are most commonly observed but IgA or IgM can also be seen.	Serum anti‐collagen VII IgG antibodies
Dermatitis herpetiformis	IgA targeting the epidermal and tissue transglutaminase	Granular deposits of IgA at the tips of epithelial papillae. Complement deposition may also occur in areas where IgA is deposited.	Autoreactive IgA targeting the epidermal and tissue transglutaminase. IgA and IgG antireticulin and antiendomysial antibodies have been identified. Additionally, these patients have a higher incidence of antinuclear and antithyroid microsomal antibodies

The diagnosis of ABDs might be difficult, and diagnostic delays are frequent for several reasons [6]. Despite some characteristic signs being identified to diagnose ABDs, such as visible bullae or vesicles, oral ulceration and desquamative gingivitis, the clinical presentation alone is insufficient for diagnosis. Furthermore, ulcers can affect any part of the oral mucosa and lack a predilection for specific areas or typical clinical characteristics. The differential diagnosis typically includes erosive oral lichen planus. Furthermore, the Nikolsky sign (i.e., the detachment of the epithelium from the underlining connective tissue with the application of gentle friction using gauze), is not always evident and is insufficient for a differential diagnosis among the different ABD subtypes. In many cases, histopathology alone may be insufficient to diagnose ABD, as it may be nonspecific, and detachment of the intraepithelial or subepithelial layers with typical acantholytic effects and subepithelial blistering might not be observed [7].

Because clinical observations and histopathological examinations are insufficient to diagnose ABDs, tissue‐bound and/or circulating autoantibodies must be detected immunologically [1]. Diagnostic procedures to confirm the clinical suspicion of ABDs include direct immunofluorescence microscopy (DIF) after mucosal biopsy, which is the most reliable method for the diagnosis of ABD. DIF is performed by analyzing antibodies chemically conjugated to fluorescent dyes [8]. Nevertheless, this technique is complicated and requires a well‐trained advanced laboratory and clinician‐laboratory coordination. Fresh specimens must be delivered immediately to the pathology laboratory, unless they are put in Michel's transport medium or solution [9]. Furthermore, the sample storage can be difficult, since fresh tissue should be immediately stored at −80°C. In addition, the laboratory processes can easily alter the autoantibodies. Pathologists experience difficulties in processing the small, friable samples under low temperatures.

More recently, circulating autoantibodies have been detected using several immunoassays, such as immunoblotting (IMB), immunoprecipitation (IMP), indirect immunofluorescence microscopy (IIF), and enzyme‐linked immunosorbent assays (ELISA) [10]. ELISAs are the most commonly used immunoassays that can measure antigens in biological samples and quantify serum autoantibody levels. This diagnostic method requires the adherence of antibodies or antigens to the surface of absorbent plates [11].

Among ABDs, SBDs comprise a heterogeneous group of illnesses in which diagnosis is challenging, and there are no definitive guidelines for an optimal diagnostic algorithm. Currently, SBD diagnosis primarily relies on a clinical correlation with histopathological observations, such as pauci‐inflammatory subepidermal cleavage or neutrophilic infiltration, and the detection of subepidermal antibodies either in situ via DIF or in the circulation using IIF and/or ELISA [12]. Furthermore, patients with pemphigus exhibit high titers of autoantibodies, whereas patients with pemphigoid have low titers of autoantibodies [13]. Additionally, the involvement of multiple antigens in pemphigoid makes the use of ELISA questionable for diagnostic purposes.

Given the absence of a consensus regarding the diagnostic concordance between ELISA and DIF, this systematic review aimed to assess the efficacy of ELISA as a diagnostic modality for SBDs and its potential as an alternative diagnostic tool in routine clinical settings. An equivalent diagnostic power of ELISA could positively impact public health and support a quicker diagnosis and less invasive method for monitoring the appropriateness of therapies in spoke hospitals. A high level of expertise is required to perform biopsies and prepare and analyze the sample using DIF. Thus, this study aimed to evaluate the performance of a more accessible and less operator‐dependent diagnostic method.

## 2 Materials and Methods

This systematic review followed the Preferred Reporting Items for Systematic Reviews and Meta‐Analyses (PRISMA) guidelines [14] and was registered in PROSPERO (registration number: CRD42022341891). This review aimed to answer the following question: “Is there diagnostic agreement for SBDs between ELISA and DIF in cohort and case‐control studies?”

This question was conceived following the PICOS model [15], as follows: patients, individuals with SBDs; intervention, ELISA; control, DIF; outcome, diagnostic agreement; and study design, cohort and case–control studies.

### 2.1 Search Strategy

A systematic literature search was performed by searching MEDLINE (PubMed), the Cochrane Database, and Scopus using a combination of free text words and MeSH terms. After several pre‐searches, the final search strategy was as follows: [(“BP180 autoantibodies” OR “pemphigoid” OR “bullous disorders” OR “autoimmune blistering diseases” OR “bullous pemphigoid” OR “subepidermal blistering diseases”) AND “enzyme linked immunosorbent assay”] OR [(“anti‐collagen VII” OR “anti‐LAD‐1” OR “laminin 332” OR “laminin 5” OR “integrin a6b4” OR “laminin g1” OR “anti‐p200”) AND “enzyme linked immunosorbent assay”].

There were no restrictions on the publication year or language, and the date of the last search was June 1, 2025. The reference lists of the included studies were cross‐checked, to identify additional studies. The following peer‐reviewed journals were manually searched for articles published between January 2003 and May 2025: *Oral Diseases*, *Clinical Oral Investigations*, *Journal of Oral Pathology and Medicine*, and *Journal of Dental Research*.

### 2.2 Eligibility Criteria

The eligibility criteria were defined by C.L. and R.P., and two reviewers (D.G. and F.S.), independently and in duplicate, screened all the articles and selected those that met the inclusion criteria. Two reviewers used specially designed forms for the screening and data extraction. Any disagreement was resolved by consulting a senior author (R.P.), until a final consensus was reached. Cohen's kappa coefficient (*k*) was used to calculate the agreement between the reviewers. The level of agreement was as follows: excellent, *k* > 0.75; fair‐to‐good, *k* of 0.40–0.75; and poor, *k* < 0.40.

### 2.3 Inclusion Criteria

Case–control or cohort studies involving both DIF and ELISA evaluations of BP180, BP230, laminin 332 (Lam332), integrin α6β4, collagen VII (COL7), laminin (LAM)‐γ1, LAD‐1, or epidermal/tissue transglutaminase in patients with bullous pemphigoid (BP), pemphigoid gestationis (PG), mucous membrane pemphigoid (MMP), epidermolysis bullosa acquisita, anti‐p200 pemphigoid, linear immunoglobulin A (IgA) disease (LABD), or dermatitis herpetiformis were included.

Only studies in which ELISA was performed at the time of first diagnosis and before the introduction of immunosuppressive therapy were included. In cases where ELISA was performed after the initiation of therapy, only data collected at diagnosis were considered.

Patients with SBDs were included in this systematic review. The diagnostic criteria for SBDs were as follows:

– Clinical presentation with signs and symptoms compatible with the diagnosis of SBD
– Histopathological examination revealed a subepidermal split with concomitant papillary dermal inflammatory cell infiltration that was predominantly eosinophilic
– DIF results showing IgG, IgA, IgM, and C3 complement deposits

### 2.4 Exclusion Criteria

Studies that evaluated the diagnostic agreement for other immunological diseases were excluded. Case reports, studies involving fewer than six participants, and animal studies were excluded. Pilot studies, studies based on novel or recombinant diagnostic tools, and studies focusing on prognosis were not included.

### 2.5 Data Extraction

Data regarding the characteristics of the included studies, including general information on the studies, number of participants, demographic characteristics, study type, and SBD features, as well as DIF and ELISA data were extrapolated and entered into a customized data collection form by two authors independently (D.G. and F.S.).

### 2.6 Quality and Cumulative Evidence Assessment

The two reviewers separately rated the risk of bias in every included study using the Newcastle‐Ottawa scale (NOS) [16]. The NOS is based on three domains: selection, comparability, and exposure. A maximum of one star is awarded for each item in the selection and exposure domains, and two stars are awarded for the comparability domain. A score of 0–3 stars was associated with a high risk of bias, 4–7 with a medium risk, and 8–9 with a low risk.

The general cumulative quality of evidence was evaluated using the Grading of Recommendations, Assessment, Development, and Evaluations (GRADE) system [17].

### 2.7 Assessment of Heterogeneity

The chi‐square test was used to evaluate whether the variation between articles was associated with heterogeneity or chance. Heterogeneity was assessed using Review Manager 5 (RevMan version 5.3.5). Cochrane's test for heterogeneity, with *p* values < 0.1 indicating significance, and the *I* ^2^ statistic, which measured inconsistency, was used to detect incongruity in the estimates of treatment effects between articles. An *I* ^2^ value > 50% indicated high heterogeneity and relevant inconsistency.

### 2.8 Data Synthesis

Meta‐analysis was performed only on homogeneous studies using Review Manager 5.3 (RevMan) software, with a significance cutoff value of *p* < 0.05. The primary outcome was the risk difference in the diagnostic yield for SBDs using ELISA compared with DIF as the gold standard.

The between‐group risk difference was evaluated using a fixed effects model or a random effects model, in the case of non‐negligible heterogeneity ( *I* ^2^ > 50%). A forest plot was used to show the meta‐analysis results, the contribution of single studies, and global estimates. The Trial Sequential Analysis software (TSA; version 0.9 beta, http://www.ctu.dk/tsa) was used to assess the power of the meta‐analysis, with an alpha error of 0.05 and a beta error of 20% [18], and to calculate the required information size (RIS), alpha‐spending function, trial sequential monitoring boundaries for benefits and harms, and futility boundaries.

## 3 Results

### 3.1 Study Selection

**FIGURE 1:**
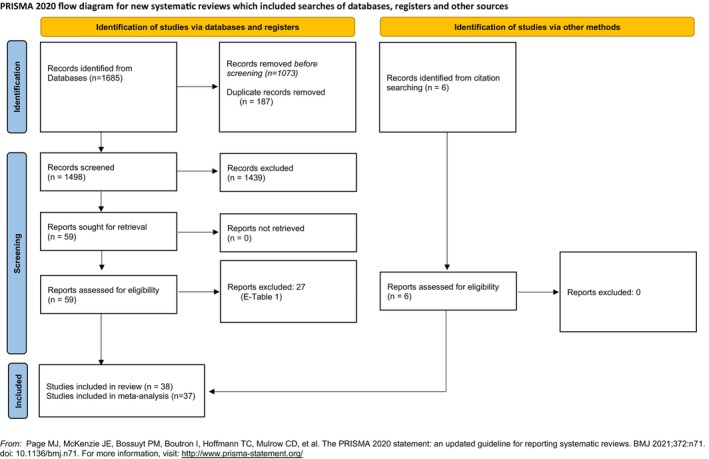
Flowchart of the selection of the studies for the review.

A flowchart of the search strategy and the respective results is shown in Figure 1. Of the 1691 titles and abstracts evaluated, 1685 were from the electronic search, and 6 were from the manual search and bibliographies of other articles. After removing 187 duplicates, 1439 articles were excluded based on a review of the titles and abstracts (inter‐reader agreement *k* = 0.81 ± 0.33). The remaining 65 articles underwent full‐text analysis, and 27 studies [19, 20, 21, 22, 23, 24, 25, 26, 27, 28, 29, 30, 31, 32, 33, 34, 35, 36, 37, 38, 39, 40, 41, 42, 43, 44, 45] were excluded (inter‐reader agreement *k* = 0.89 ± 0.21) (E‐Table 1 in Supporting Information). Finally, 38 studies fulfilled the study criteria and were included in the systematic review [46, 47, 48, 49, 50, 51, 52, 53, 54, 55, 56, 57, 58, 59, 60, 61, 62, 63, 64, 65, 66, 67, 68, 69, 70, 71, 72, 73, 74, 75, 76, 77, 78, 79, 80, 81, 82, 83]. Among the included papers, 37 were sufficiently homogeneous for quantitative synthesis. The study by Groth et al. [64] was not included in the meta‐analysis, as it was the only study evaluating the LAM‐γ1 antigen.

### 3.2 Study Characteristics

Among the included studies, 24 were prospective, and 14 were retrospective. The majority of these studies were conducted in Europe in a hospital setting, and their time frames ranged from 1997 to 2022. The characteristics of the included studies are shown in Table 2.

**TABLE 2:** Summary of the major characteristics of each included study.

Author and Year	Study design	Total sample of patients and respective disease	Resume	Percentage of diagnostic agreement	Manufacture	Country	Setting	Time of testing
Barnadas 2008 [46]	R	28 (24 BPD+4PG)	Patients received a diagnosis based on DIF. BP180 ELISA was positive in 26/28 patients. In particular it was positive in 22/24 BPD patients and in 4/4 PG patients	BP180 = 93% (92% for BPD patients and 100% for PG patients)	MBL Co Ltd., Nagoya, Japan.	Spain	Dermatology dep. of Sant Pau Hospital	Before the initiation of pharmacological treatment
Bernard 2013 [47]	R	154 MMP	Patients with positive results of DIF were included. Their serum samples were tested: 60/154 were positive for BP180‐NC16A ELISA, 16/154 samples had positive BP230 ELISA scores, and 31/154 samples had positive Lam332 ELISA scores.	BP180 = 39% BP230 = 10.4% Lam332 = 20.1%	MBL Co Ltd. Nagoya, Japan.	France	Four French dermatology dep. for autoimmune bullous diseases	Before the initiation of pharmacological treatment
Bing 2015 [48]	P	37 BPD	DIF and ELISA were performed. DIF was positive in all patients. The anti‐BP180 NC16A IgE autoantibody titers were detected in the serum or blister fluid in 27/37 patients.	BP180 = 73%	MBL Co Ltd. Nagoya, Japan.	China	Four dermatology dep. Beijing and Cangzhou Hospitals	Before the initiation of pharmacological treatment
Chanprapaph 2021 [49]	P	68 BPD	All cases showed positivity on DIF. Among them, 33 patients were positive to anti‐BP180 IgG and anti‐BP230 IgG ELISAs, whereas 14 ones were positive to anti‐BP180 IgG alone, and 5 to anti‐BP230 IgG alone.	BP180 = 69% BP230 = 56%	Commercial Elisa kits Euroimmun AG, Lubecca, Germany	Thailand	Dermatology dep. of Ramathibodi and Mahidol Hospitals	Before the initiation of pharmacological treatment
Charneux 2011 [50]	R	138 BPD	Patients had been diagnosed by direct IF. Among them, 119 had a positive BP180 ELISA result and 81 had a positive BP230 ELISA result.	BP180 = 86% BP230 = 59%	MBL Co Ltd., Nagoya, Japan	France	French academic dermatology dep.	Before the initiation of pharmacological treatment
Cozzani 2020 [51]	R	54 LABD	Sera were collected from patients who demonstrated linear IgA‐deposit by DIF. BP180‐NC16A ELISA results were positive in 17/54 patients and BP230 ELISA results were positive in 14/54 patients.	BP180 = 32% BP230 = 26%	MBL Co Ltd. Nagoya, Japan.	Italy	Five different Italian clinical centers	Before the initiation of pharmacological treatment
Cozzani 2016 [52]	R	78 MMP	Patients were diagnosed by DIF. BP180 enzyme‐linked immunoassay (ELISA) was positive in 26 patients; 9 patients were positive by BP230 ELISA and 3 by collagen VII ELISA.	BP180 = 33% BP230 = 11.5% ColVII = 3.8%	MBL Co Ltd. Nagoya, Japan.	Italy	Five different Italian clinical centers	Before the initiation of pharmacological treatment
Dart 2021 [53]	P	49 MMP	DIF showed positive results in all patients. BP180 ELISA was positive in 12 patients out of 49, 6 patients were positive by BP230 ELISA and 3 by Laminin 332.	BP180 = 24% BP230 = 12% Lam332 = 6%	MBL Co Ltd. Nagoya, Japan.	United Kingdom	Eyes, oral and dermatology clinics of London	Before the initiation of pharmacological treatment
Dikmen 2022 [54]	P	499 BPD	Patients' diagnosis was made by DIF and ELISA. 369 patients had a positive BP180 NC16A ELISA value. 205 patients had a positive BP230 ELISA value.	BP180 = 74% BP230 = 41%	Euroimmun, Lubeck, Germany and MBL, Nagoya, Japan	Germany Hungary Finland France Bulgaria Greece	Sixteen different European dermatology dep.	Before the initiation of pharmacological treatment
Esmaili 2015 [55]	P	50 BPD	Patients were diagnosed by DIF. Among them, 44/50 patients had a positive BP180 ELISA value. 24/50 had a positive BP230 ELISA value.	BP180 = 88% BP230 = 48%	Euroimmun AG, Lubeck, Germany.	Iran	Razi Dermatology Hospital Tehran	Before the initiation of pharmacological treatment
Fania 2012 [56]	P	26 BPD	Immunoserological characteristics of all patients were DIF+ with IgG against the NH2‐terminus and COOH terminus of BP230 in 19/26 and IgG against the NH2‐terminus and COOH‐terminus of BP180 in 22/26.	BP180 = 85% BP230 = 74%	N/D	Germany and Italy	N/D	Before the initiation of pharmacological treatment
Feliciani 2009 [57]	P	30 PD	DIF was performed and showed positivity in all patients. Patients IgG reactivity was tested by ELISA. IgG against BP180 and BP230 was found in 14 patients, against BP180 only in 9 patients and against BP230 only in 7 patients.	BP180 = 77% BP230 = 70%	N/D	Germany and Italy	N/D	Before the initiation of pharmacological treatment
Gasparini 2022 [58]	P	31 MMP	The diagnosis in patients was established by DIF. Anti‐BP180 IgG antibodies were detected in 12/31 cases and anti‐BP230 IgG antibodies were detected in only 1/31 cases.	BP180 = 39% BP230 = 3%	MBL Co Ltd. Nagoya, Japan.	Italy	Dermatology dep. of five different Italian hospitals	Before the initiation of pharmacological treatment
Giusti 2018 [59]	R	71 BPD	DIF and ELISA test were performed. All patients were DIF+. ELISA BP180 was positive in 59/71 and ELISA BP230 in 33/71.	BP180 = 83% BP230 = 46%	MBL Co Ltd. Nagoya, Japan.	France	Dermatology dep. of Reims Hospital	Before the initiation of pharmacological treatment
Gornowicz‐Porowska 2022 [60]	P	15 MMP	DIF and ELISA tests for anti‐BP180/BP20 IgG antibodies were performed. Among them, 13/15 resulted positive at DIF, while 9/15 resulted positive at ELISA.	BP180 = 60%	Euroimmun ELISA kits (Luebeck, Germany)	Poland	Dermatology dep. of the Poznan's hospital	Before the initiation of pharmacological treatment
Gornowicz‐Porowska 2017a [61]	R	82 BPD	Patients were diagnosed by DIF and ELISA tests. Target antigens (BP180, BP230) detected with ELISAs were found in 80 patients (60 only BP180; 2 only BP230 and BP 180/230 in 18).	BP180 = 97,5% BP230 = 25%	Euroimmun ELISA kits (Luebeck, Germany)	Poland	Dermatology dep. of the Poznan's hospital	Before the initiation of pharmacological treatment
Gornowicz—Porowska 2017b [62]	P	37 BPD	DIF was performed and showed positivity in all patients. Diagnosis was confirmed by positive BP180 and/or BP230 IgG ELISA in serum samples. Results of ELISA were positive in 36/37. BP230 results were positive in 9 of 33 patients.	BP180 = 97,3%	Euroimmun ELISA kits (Luebeck, Germany)	Poland	Dermatology dep. of the Poznan's hospital	Before the initiation of pharmacological treatment
Grootenboer‐Mignot 2010 [63]	P	78 BPD	All selected patients were DIF positive. Of these, 32/78 had a positive BP180 ELISA result while 41/78 had a positive BP230 ELISA result.	BP180 = 41% BP230 = 52,5%	MBL Co Ltd. Nagoya, Japan.	France	N/D	Before the initiation of pharmacological treatment
Groth 2011 [64]	P	35 anti‐p200 pemphigoid	All patients were DIF+. Sera were tested with ELISA using human laminin γ1. ELISA was positive in 24/35 patients selected.	LAM γ1 = 69%	N/D	Germany	Dermatology dep. Lübeck	Before the initiation of pharmacological treatment
Hoffman 2002 [65]	P	116 BPD	All patients showed positive reactivity by DIF. ELISA was performed and 108/116 resulted positive.	BP180 = 93.1%	N/D	Germany	Dermatology dep. of FAU Erlangen‐Nürnberg	Before the initiation of pharmacological treatment
Keller 2016 [66]	R	170 BPD	DIF and ELISA were performed. All patients showed positivity by DIF. BP180 ELISA results were positive in 92/170 patients and BP230 ELISA results were positive in 82/170 patients.	BP180 = 54.1% BP230 = 48.2%	MBL Co Ltd. Nagoya, Japan.	USA	Dermatology dep., UH CASE Medical Center	Before the initiation of pharmacological treatment
Kromminga 2004 [67]	P	56 BPD	DIF and ELISA tests were carried out. DIF was positive for all the patients, while ELISA BP230 was positive for 35/56 patients.	BP230 = 63%	N/D	Germany	N/D	Before the initiation of pharmacological treatment
Lee 2012 [68]	P	47 BPD	Patients showed positive reactivity by DIF. Serum IgG levels of BP180 and BP230 autoantibodies were evaluated using ELISA. BP180 ELISA was positive in 46 patients. 34 were positive for BP230 ELISA.	BP180 = 98% BP230 = 72.3%	MBL Co Ltd. Nagoya, Japan.	Korea	Dermatology dep. Yonsei, College of Medicine.	Before the initiation of pharmacological treatment
Li 2022 [69]	R	164 BPD	Patients were tested by DIF and BP180 ELISA. DIF was found positive in 128/164 patients. Of all 154 showed positivity to BP 180 ELISA.	BP180 = 93%	N/D	China	Chinese academy of medical sciences	Before the initiation of pharmacological treatment
Mariotti 2004 [70]	P	78 BPD	Serum samples were obtained from 78 patients. The clinical diagnosis of BPD was confirmed by DIF. All patients underwent BP180 NC16A ELISA test. IgG reactivity was detected in 64/78 BPD sera.	BP180 = 82%	N/D	Italy	Immunology and Allergology dep. of San Gallicano Hospital	Before the initiation of pharmacological treatment
Messingham 2009 [71]	P	43 BPD	Serum samples were collected from patients with a confirmatory DIF positivity. All sera were tested by ELISA for the presence of IgG specific for BP180. IgG were found in 35/43 patients.	BP180 = 81%	MBL Co Ltd. Nagoya, Japan.	USA	Dermatology dep., University of Iowa	Before the initiation of pharmacological treatment
Patsatsi 2008 [72]	P	13 BPD	Patients were enrolled for DIF and ELISA tests. All patients were DIF+. Anti‐BP180 NC16A antibodies were detected by ELISA in 9/13 patients. Anti‐BP230 antibodies were detected by ELISA in 8/13 patients.	BP180 = 69% BP230 = 61.5%	MBL Co Ltd. Nagoya, Japan.	Greece	Dermatology dep., Aristotle University, Thessaloniki.	Before the initiation of pharmacological treatment
Petruzzi 2021 [73]	P	30 BPD	Patients were tested by DIF and ELISA. All patients were positive to DIF. 15 patients resulted positive to BP180‐NC16A ELISA test.	BP180 = 50%	MBL Co Ltd. Nagoya, Japan.	Italy	Department of Oral Medicine of University “Aldo Moro”. Bari	Before the initiation of pharmacological treatment
Sakuma‐Oyama 2004 [74]	P	102 BPD	Patients were assayed by BP180‐NC16a ELISA before diagnosis received by DIF. 91/102 sera exhibited IgG reactivity to the NC16a of BP180.	BP180 = 89%	MBL Co Ltd. Nagoya, Japan.	UK	Four English hospitals of London	Before the initiation of pharmacological treatment
Sernicola 2019 [75]	P	13 BPD	Blister fluid was collected from each of the 13 individuals before initiating treatment and all sera underwent to ELISA test. BP180 Elisa test was positive in 10/13 patients.	BP180 = 77%	MBL Co Ltd. Nagoya, Japan.	Italy	Dermatology dep., University of Padua	Before the initiation of pharmacological treatment
Sitaru 2007 [76]	R	118 BPD	BP180 NC16A ELISA test was performed in patients diagnosed with DIF. ELISA resulted positive in 105/118 patients.	BP180 = 90%	MBL Co Ltd. Nagoya, Japan.	Germany	Two dermatologic dep. of Lubeck and Wurzburg.	Before the initiation of pharmacological treatment
Tampoia 2009 [77]	P	20 BPD	Of all patients with DIF+ ELISA tests were conducted, in 18/20 patients' sera showed IgG reactivity to the NC16a of BP180.	BP180 = 90%	MBL Co Ltd. Nagoya, Japan.	Italy	Dermatology dep. of University of Bari	Before the initiation of pharmacological treatment
Tani 2015 [78]	R	25 PG	Patients received diagnosis after clinical exam and DIF. ELISA test was performed, showing positive results in 23/25 patients for BP180 and in 4/25 patients for BP230, respectively.	BP180 = 92% BP230 = 16%	MBL Co Ltd. Nagoya, Japan.	Japan	Dermatology dep., Kurume University School of Medicine	Before the initiation of pharmacological treatment
Terra 2011 [79]	R	8 MMP	Diagnosis was based on clinical examination and by DIF. The latter was positive in 7/8 patients. ELISA LN‐332 was performed in 8 patients and resulted positive in 6/8.	Lam 332 = 75%	N/D	Netherland	Three dep. of University Medical Center	Before the initiation of pharmacological treatment
Treco 2023 [80]	R	26 BPD	Patients were positive to DIF and ELISA test was performed. Evaluation by ELISA detected that 24/26 were positive for BP180‐specific IgG and 14/26 had BP230‐specific IgG.	BP180 = 92% BP230 = 54%	MBL Co Ltd. Nagoya, Japan.	USA	Dermatology dep. University of Iowa.	Before the initiation of pharmacological treatment
Van Beek 2022 [81]	R	154 MMP	Patients, after diagnosis with DIF, underwent Elisa test. BP180 antigen was detected in 90/154 patients, followed by laminin 332 antigen in 13/154 patients and BP230 in 3/154 patients, respectively.	BP180 = 58% BP230 = 1.9% Lam332 = 8.4%	N/D	Germany	Two German dep. Lübeck and TU Dresden Universities.	Before the initiation of pharmacological treatment
Yang 2012 [82]	P	62 BPD	DIF and ELISA test were performed in all included patients. All patients were diagnosed by DIF. BP180‐NC16a ELISA was positive in 54/62 patients. BP230 ELISA was positive in 35/62.	BP180 = 87.1% BP230 = 56.5%	MBL Co Ltd. Nagoya, Japan.	China	Dermatology and Venereology dep. of Shandong	Before the initiation of pharmacological treatment
Zillikens 1997 [83]	P	50 BPD	DIF and ELISA test were performed. 29/50 BP180 were positive in the NC16A ELISA. 30/50 contained autoantibody reactivity to the BP230 antigen.	BP180 = 58% BP230 = 60%	N/D	USA	Dermatology dep., Milwaukee, Wisconsin	Before the initiation of pharmacological treatment

Abbreviations: BPD, bullous pemphigoid disease; Dep., department; LABD, linear immunoglobulin A (IgA) bullous dermatosis; MMP, mucous membrane pemphigoid; *P*, prospective; PD, pruritic dermatose; PG, pemphigoid gestation; *R*, retrospective.

Of the included studies, 27 involved patients with BP, 7 involved patients with MMP, and 1 involved patients with LABD, anti‐p‐200 pemphigoid, PG, and pruritic dermatoses (PD).

For all included patients, the diagnosis of SBDs was confirmed using DIF.

ELISAs were conducted for the molecular identification of target antigens in SBD, as follows: against BP180 in 35 studies, against BP230 in 27 studies, against Lam332 in 4 studies, and against COL7 and the LAM‐γ1 antigen in 1 study.

### 3.3 Quality Assessment (GRADE) and Risk of Bias in Individual Studies

**FIGURE 2:**
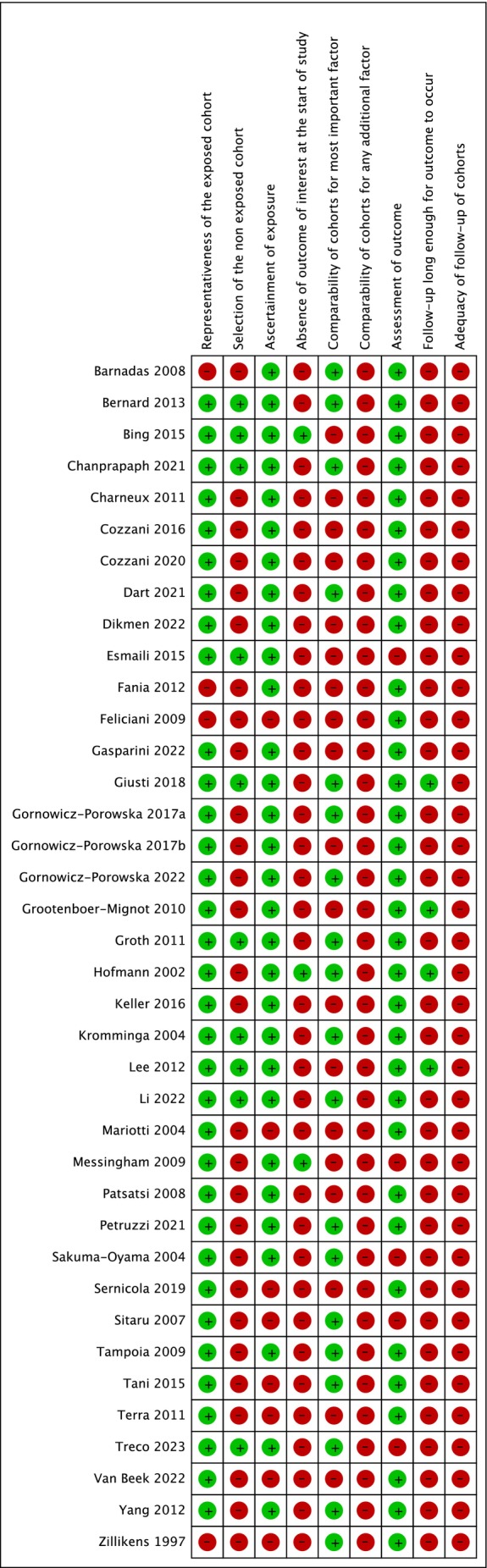
Risk of bias graph.

**FIGURE 3:**
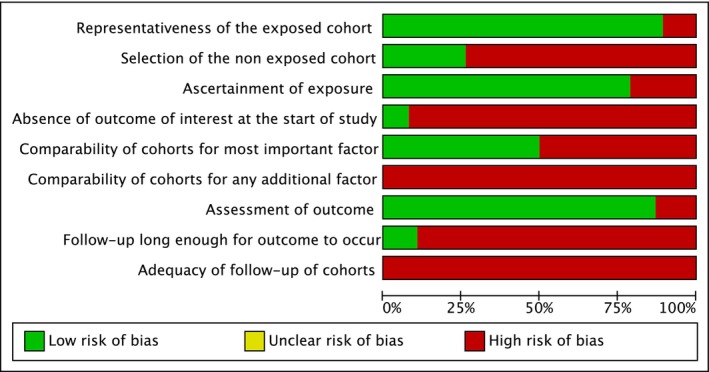
Risk of bias summary.

Among the included studies, 21 had a high risk of bias [46, 50, 51, 52, 54, 55, 56, 57, 58, 62, 66, 71, 72, 73, 75, 76, 77, 79, 80, 81], and 17 had a medium risk [47, 48, 49, 53, 59, 60, 61, 63, 64, 65, 67, 68, 69, 70, 74, 78, 82, 83] (Figures 2 and 3). None of the studies exhibited a low risk of bias.

The methodological strength of the evidence and certainty of the conclusions of the meta‐analyses were evaluated using GRADE (Table 3). The GRADE summary reported that the meta‐analysis results should be interpreted with high caution, owing to the high risk of bias in a significant number of included studies and the general inconsistency and imprecision of the reported results.

**TABLE 3:** GRADE summary of findings for meta‐analysis on effectiveness of ELISA test as a diagnostic tool for SBD.

Quality assessment, outcome: Effectiveness of ELISA test as a diagnostic tool for main SBD						
Question: Could be ELISA test used as a diagnostic examination for main SBD in daily clinical practice?						
Number of studies according to each meta‐analysis	Study design	Risk of bias	Inconsistency	Indirectness	Imprecision	Publication bias
Meta‐analysis evaluating BP 180 35 (Figure 4)	Retrospective and prospective studies	Serious	Serious^a^	Not serious	Serious^b^	Undetected
Meta‐analysis evaluating BP 230 27 (Figure 5)	Retrospective and prospective studies	Serious	Serious^a^	Not serious	Serious^b^	Undetected
Meta‐analysis evaluating Lam 332 4 (Figure 6)	Retrospective and prospective studies	Serious	Serious^a^	Not serious	Serious^b^	Undetected
Meta‐analysis evaluating BPD 27 (Figure 7)	Retrospective and prospective studies	Serious	Serious^a^	Not serious	Serious^b^	Undetected
Meta‐analysis evaluating MMP 7 (Figure 8)	Retrospective and prospective studies	Serious	Serious^a^	Not serious	Serious^b^	Undetected

^a^ Due to high heterogeneity across studies.

^b^ Due to wide confidence intervals between studies.

### 3.4 Results of the Meta‐Analysis and Trial Sequential Analysis

Meta‐analyses were conducted to evaluate the agreement between ELISA and DIF results in patients with BP and MMP. Additional meta‐analyses were conducted to evaluate the concordance between the BP180, BP230, and Lam332 antigens and DIF results in patients with SBDs, including BP, PG, MMP, epidermolysis bullosa acquisita, anti‐p200 pemphigoid, LABD, and dermatitis herpetiformis.

#### 3.4.1 BP

**FIGURE 4:**
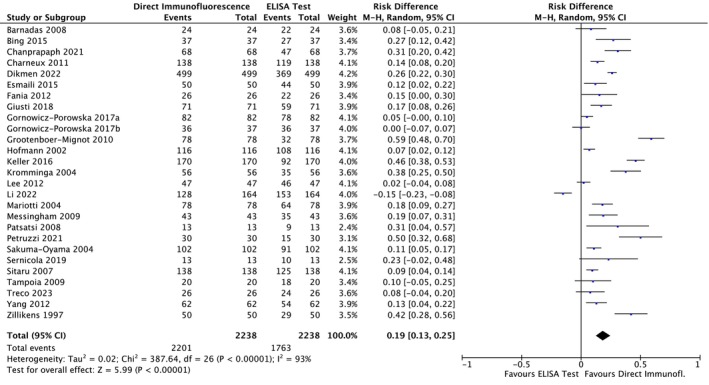
Forest plot of comparison of diagnostic agreement in patients affected by bullous pemphigoid between direct immunofluorescence (DIF) and ELISA.

**FIGURE 5:**
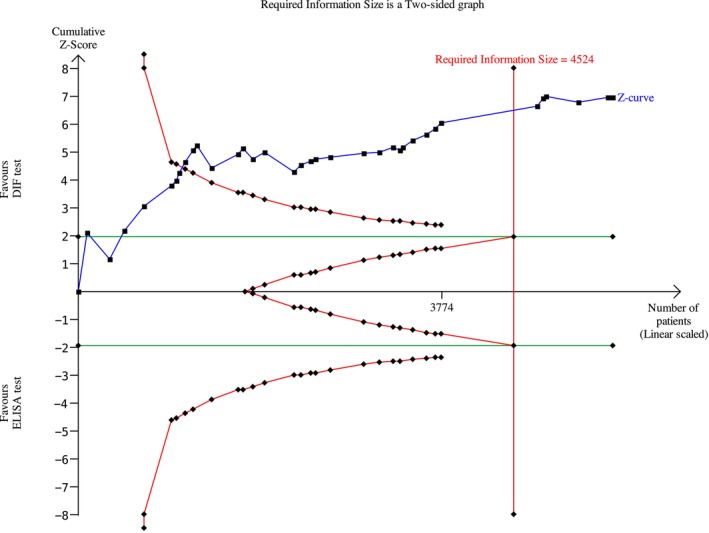
Trial sequential analysis (TSA) of diagnostic agreement of SBD between DIF and ELISA test regarding BP 180.

**FIGURE 6:**
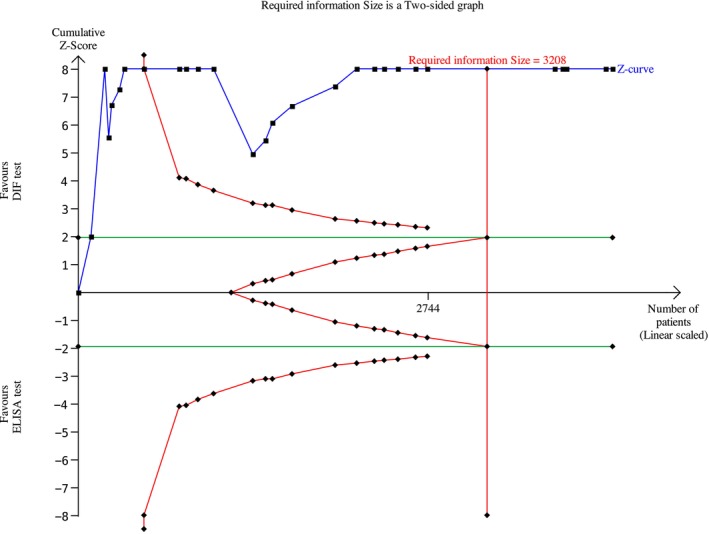
Trial sequential analysis (TSA) of diagnostic agreement of SBD between DIF and ELISA test regarding BP 230.

**FIGURE 7:**
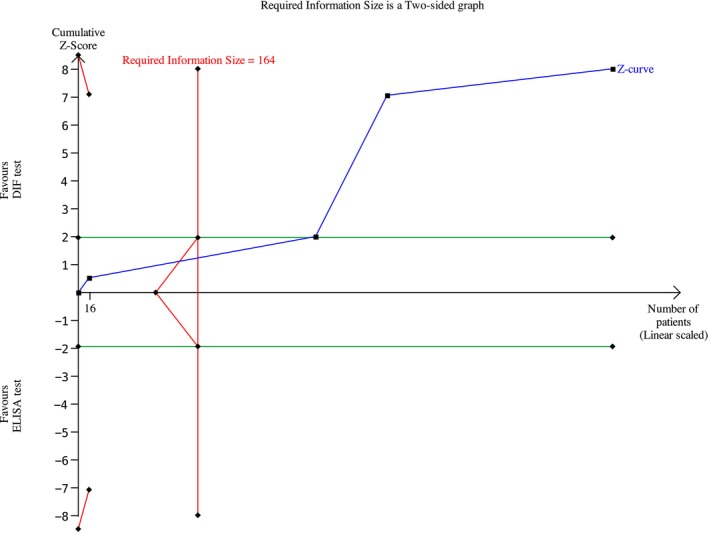
Trial sequential analysis (TSA) of diagnostic agreement of SBD between DIF and ELISA test regarding Lam 332.

**FIGURE 8:**
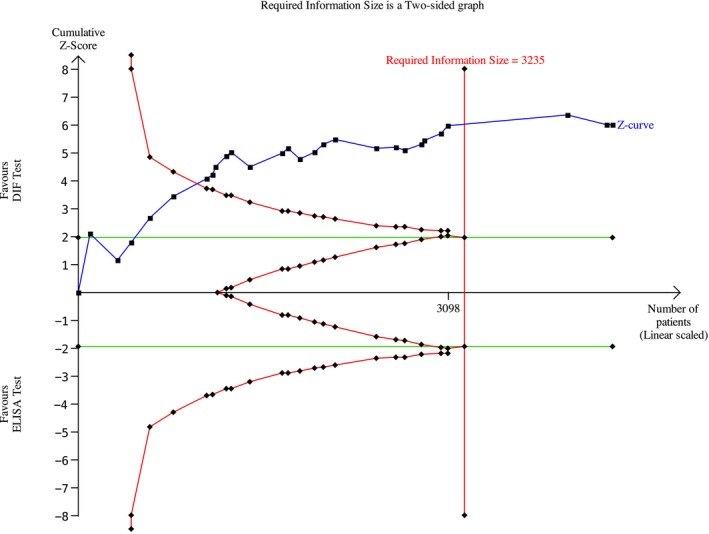
Trial sequential analysis (TSA) of diagnostic agreement of bullous pemphigoid between DIF and ELISA tests.

**FIGURE 9:**
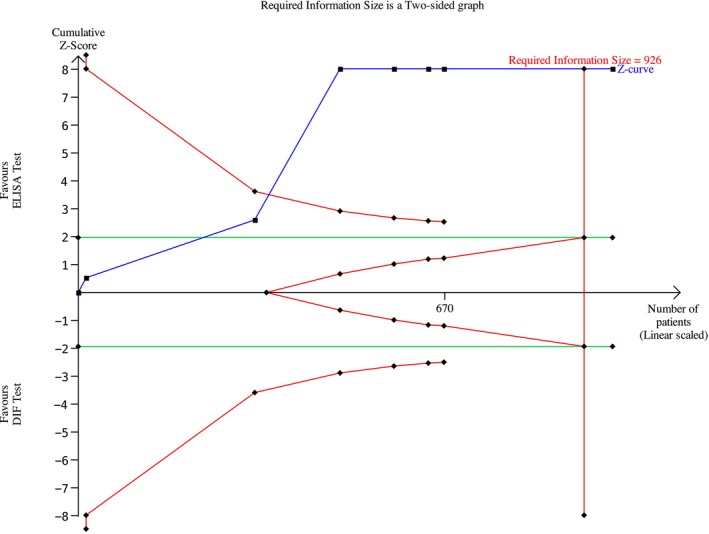
Trial sequential analysis (TSA) of diagnostic agreement of mucous membrane pemphigoid between DIF and ELISA tests.

Among the BP studies, 27 were included in the meta‐analysis; thus, 2238 patients were analyzed. A meta‐analysis was conducted using a random effects model owing to the non‐negligible heterogeneity across studies. The risk difference was 0.19 (95% CI: 0.13–0.25; *p* < 0.00001) for BP (Figure 4) The evidence retrieved was confirmed by the TSA analyses, which depicted that all *z*‐curves crossed the conventional boundary and reached the alpha‐spending function and RIS threshold, thus giving high reliability to the quantitative analyses (Figures 5, 6, 7, 8, 9).

#### 3.4.2 MMP

**FIGURE 10:**
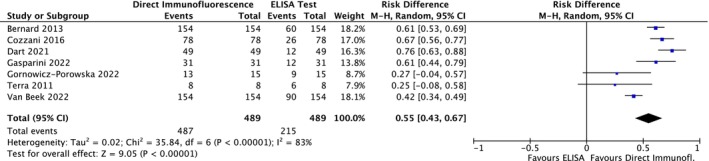
Forest plot of comparison of diagnostic agreement in patients affected by mucous membrane pemphigoid between direct immunofluorescence (DIF) and ELISA.

Among the MMP studies, 7 were included; thus, 489 patients were included in the meta‐analysis. The meta‐analysis was conducted using a random effects model, because the heterogeneity across studies was non‐negligible. The risk difference was 0.55 (95% CI: 0.43–0.67; *p* < 0.00001) for MMP (Figure 10). The evidence retrieved was confirmed by the TSA analyses, which showed that all *z*‐curves crossed the conventional boundary and reached the alpha‐spending function and RIS threshold, thus giving high reliability to the quantitative analyses (Figures 5, 6, 7, 8, 9).

### 3.5 Concordance Between BP180, BP230, and Lam332 Antigen and DIF Results

Regarding specific antigens identified using ELISAs, 35 studies with 2776 patients involved BP180, 27 studies with 2097 patients involved BP230, and 4 studies with 365 patients involved Lam332. Thus, three different meta‐analyses were conducted among the BP180, BP230, and Lam332 antigens. Given the high heterogeneity among the studies, a random effects model was used in all meta‐analyses.

**FIGURE 11:**
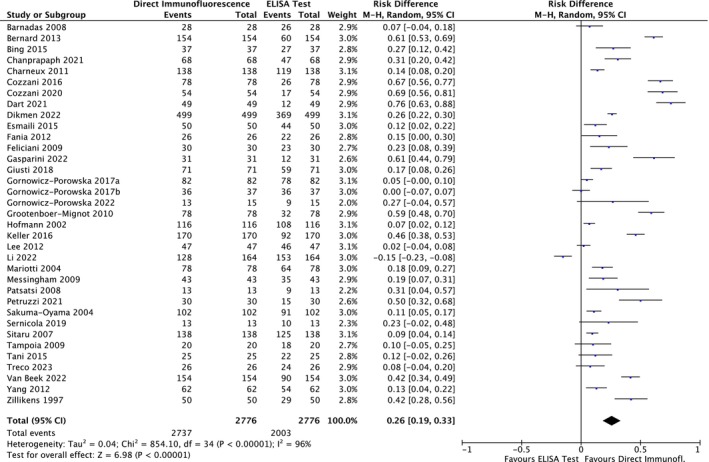
Forest plot of comparison of diagnostic agreement regarding BP 180 in patients affected by SBD between DIF and ELISA.

Regarding the BP180 antigen, the meta‐analysis showed a risk difference of 0.26 (95% CI: 0.19–0.33; *p* < 0.00001) (Figure 11). Consequently, the risk of missing a diagnosis using an ELISA for BP180 increased by 26% compared to using DIF.

**FIGURE 12:**
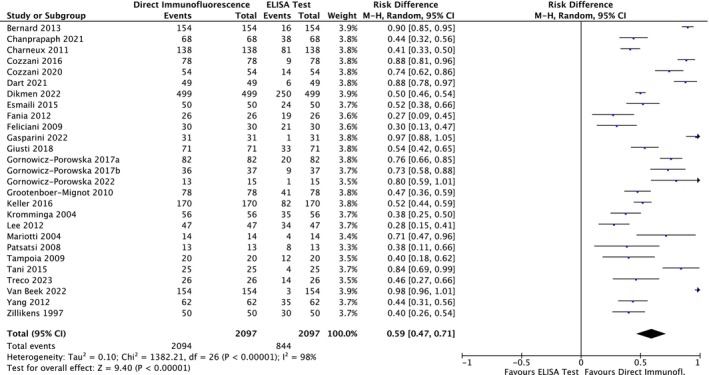
Forest plot of comparison of diagnostic agreement regarding BP 230 in patients affected by SBD between DIF and ELISA.

Regarding the BP230 antigen, the meta‐analysis showed a risk difference of 0.59 (95% CI: 0.47–0.71; *p* < 0.00001) (Figure 12). Consequently, the risk of missing a diagnosis using an ELISA for BP230 increased by 59% compared to using DIF.

**FIGURE 13:**
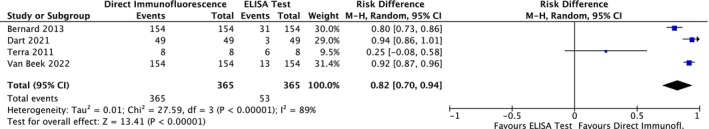
Forest plot of comparison of diagnostic agreement regarding Lam 332 in patients affected by SBD between DIF and ELISA.

Regarding the Lam332 antigen, the meta‐analysis showed a risk difference of 0.82 (95% CI: 0.70–0.94; *p* < 0.00001) (Figure 13). Consequently, the risk of missing a diagnosis using an ELISA for Lam332 increased by 82% compared to using DIF.

## 4 Discussion

This review aimed to determine whether ELISA can provide diagnostic capabilities equivalent to those of DIF, potentially justifying the replacement of DIF with ELISA for the diagnosis of SBDs.

This systematic review revealed that ELISA has a high risk of false negatives in patients affected by BP and MMP (19% and 55%, respectively; *p* < 0.05). All meta‐analyses confirmed the inferiority of ELISA as a diagnostic tool for SBD when compared with DIF, even when a random effects model was used due to the high heterogeneity among the included studies. This heterogeneity can be resolved by designing studies with more homogenous diagnostic criteria and laboratory data interpretation. However, the results of the present analysis must be interpreted with caution, since more than half of the studies were assessed as having a high risk of bias. Moreover, the small number of studies included in each meta‐analysis had wide confidence intervals, indicating possible imprecision. Nevertheless, the TSA revealed that the *z*‐curves crossed the conventional boundary and reached the alpha‐spending function and RIS threshold. Therefore, based on these data, DIF should still be considered the gold standard method for the diagnosis of the diseases analyzed in this review.

A previous systematic review by Van de Gaer et al. [84] compared the sensitivity and specificity of ELISA with those of DIF in both IBDs and SBDs and found that ELISA can support but cannot replace DIF. Conversely, a systematic review by Tampoia et al. [77] revealed a higher specificity and sensitivity of ELISA for detecting anti‐BP180 autoantibodies (0.98 and 0.87, respectively). This discrepancy may be related to the different outcomes of the reviews and less strict inclusion criteria. In fact, we included only patients who had a DIF‐confirmed diagnosis and who did not receive immunosuppressive treatment.

The results of this review generated some concerns. The first concern is a technical laboratory issue. ELISAs are extensively used to detect autoantibodies targeting specific autoantigens [83]. Approximately 85%–96% of autoantibodies in BP can be detected using commercially available COL17‐NC16A ELISA or chemiluminescence enzyme immunoassay (CLEIA) systems [85, 86, 87]. However, the antigens targeted by these ELISA/CLEIA systems are limited to specific domains of the basement membrane zone (BMZ) proteins, such as COL17 (NC16A), BP230 (N/C‐terminus), and COL7 (NC1/NC2). Due to the low titers and heterogeneity of MMP autoantibodies, only 30%–52% of autoantibodies in COL17‐type MMP can be detected using the COL17‐NC16A ELISA/CLEIA [88, 89]. Consequently, a significant proportion of patients with MMP remain undiagnosed or untreated. Moreover, not all antigens involved in the pathogenesis of SBDs are known. This could partly explain the number of false negatives for the most well‐known antigens. DIF is characterized by contact of the samples with reagents, followed by the placement of a glass cover on the slides. DIF is a sensitive and specific laboratory method that utilizes fluorescently labeled antibodies to detect the presence and distribution of specific antigens in tissue samples. In ABDs, the target antigen is the crystallizable fragment (Fc) portion of immunoglobulin (Ig). Because the Fc domain is conserved within a species, a labeled antibody can be used to detect any autoantibody, regardless of the primary antigen, even if unknown [90]. Currently, this is the most reliable method for diagnosing ABDs [91].

Second, the distinctive antigenic profiles of various SBDs must be considered. A specific blistering disease can express multiple antigens; for example, BP can show positivity for both BP130 and BP230 (Table 1). Therefore, different clinical features are configured based on the antigenic profile of the disease. MMP is caused by autoantibodies targeting autoantigens within the BMZ, such as collagen XVII (COL17, also known as BP180), BP230, Lam332, integrin α6/β4, and COL7 [92]. Among these, the C‐terminus of COL17 and Lam332 is considered major autoantigens for MMP. Autoantibodies against integrin α6/β4 have been associated with the development of ocular lesions. Mutations in *BP180* are associated with a blistering disorder known as junctional epidermolysis bullosa, emphasizing the role of BP180 in maintaining skin integrity [93]. BP180 is the primary target in BP, a common ABD that primarily affects the skin without significant scarring [94]. Epitope analysis of BP180 has revealed correlations between different epitopes and the involvement of skin versus mucosal tissues, as well as the presence or absence of scarring [94]. Understanding these correlations could guide early treatment decisions in patients with MMP, particularly those with exclusive oral involvement. Another important antigenic profile is represented by the anti‐Lam332 within the SBD, which has been associated with an increased risk of malignant neoplasms compared to the general population (relative risk: 6.8; 95% CI: 3.3–12.5). DIF analysis of different SBDs demonstrates similar results of linear binding of IgG, IgA, and/or C3 at the BMZ. Thus, performing a differential diagnosis among SBDs is not always suitable. ELISA represents an important tool for differential diagnosis and determining the prognosis of patients.

The third consideration concerns the quantification of disease severity. Several studies have suggested that ELISA can be used to monitor antibody titers [95]. Autoantibody titers are closely related to disease activity and could, in some, but not all patients, therefore be used to predict relapses and remissions [96]. Recent studies indicate that antibody titers are correlated with disease activity, which is clinically useful, especially for pemphigus. Furthermore, blood sampling to perform ELISAs is a well‐tolerated, nonsurgical procedure and routine practice for the patient. This evaluation cannot be conducted using DIF analysis. However, surrogate parameters have been identified to evaluate disease severity. In some included studies, patients were classified using different methods to rank “disease severity,” including the number of blisters/erosions [76]; clinical criteria, including an age > 70 years, lack of scarring, and absence of head, neck, or mucosal involvement [66]; or the Oral Disease Severity Score (ODSS) [73]. In contrast, Tampoia et al. [77] calculated the sum of the scores for activity, extent, and pruritus. A validated standardized scale of severity is needed, and parameters that correlate disease severity with antibody titers are lacking. A summary of the severity scores of the main diseases is presented in E‐Table 2.

Despite the accurate screening process, this systematic review had some limitations. The main limitations were the high heterogeneity in the follow‐up duration and methodology of the included studies, cutoff values used to validate the ELISAs, and choice of controls, with some studies using healthy patients and others using patients affected by other autoimmune diseases. These conditions do not guarantee a clear interpretation of the results. Regarding the external validity of the results, a high level of expertise is necessary to perform biopsies and prepare and analyze samples using DIF. This level of expertise was not required for the ELISA. Evidence of the superior or at least equivalent diagnostic power of ELISAs could have positively impacted public health by leading to a faster diagnosis and less invasive method for therapeutic monitoring in spoke hospitals. Diagnostic agreement between DIF and ELISAs could have indicated that ELISAs represented an easier and quicker method for clinicians, since early recognition allowing timely therapy is essential. Currently, the correct approach to the diagnosis of SBDs involves both techniques: DIF represents the gold standard for diagnosis, and ELISAs can aid in the differential diagnosis and disease monitoring during follow up.

## 5 Conclusions

This meta‐analysis concluded that ELISAs cannot be regarded as a gold standard equivalent to DIF for the diagnosis of SBDs. Despite these limitations, ELISAs remain a valuable diagnostic tool, particularly in settings with limited access to advanced immunofluorescence techniques or specialized dermatopathologists. In hospitals without expertise in DIF or where DIF is not readily available, ELISAs may serve as a practical alternative for the initial screening and guide clinicians toward a suspected diagnosis, allowing the timely initiation of treatment. Additionally, their ease of use, low cost, and noninvasive nature make it a suitable option for the routine monitoring of disease activity and responses to therapy.

## Funding

The authors have nothing to report.

## Conflicts of Interest

The authors declare no conflicts of interest.

## Supporting information


**Table S1:** Studies excluded with reason after full text evaluation.

**Table S2:** A summarized scoring systems of diseases severity.

## Data Availability

The data that support the findings of this study are available from the corresponding author upon reasonable request.
