# Supplementary material for: Diagnostic Agreement in Subepidermal Blistering Diseases: Is ELISA Test Reliable as Direct Immunofluorescence? A Systematic Review, Meta‐Analysis, and Trial Sequential Analysis
Source: J Oral Pathol Med. 2025 Dec 12;55(4):391–418. doi: 10.1111/jop.70088 (PMC13065897; doi:10.1111/jop.70088)
Supplement: Supplementary file 1 — Table S1: Studies excluded with reason after full text evaluation. [file JOP-55-391-s002.docx]

**E-Table 1.** Studies excluded with reason after full text evaluation.

| **Author** | **Reason of exclusion** |
| --- | --- |
| Amo et al. (19) | No ELISA performed. |
| Chiavérini et al. (20) | Small number of patients in the study. |
| Csorba et al. (21) | This study evaluated the reactivity of IgA autoantibodies with a recombinant diagnostic tool. |
| Damoiseaux et al. (22) | Results regarding BP180 and BP230 were not presented separately. |
| Daneshpazhooh et al. (23) | The authors did not compare DIF and ELISA test. |
| Desai et al. (24) | DIF was not performed. |
| Didona et al. (25) | Number of ELISA tests was not reported. |
| Di Zenzo et al. (26) | Measures were performed by different laboratories. |
| Döpp et al. (27) | The authors did not specify the number of participants of each group. |
| Fairley et al. (28) | The study was focused on patients negative to Elisa Test. It was not clearly specified the number of patients positive to DIF. |
| Goyal et al. (29) | DIF was not performed. |
| Feng et al. (30) | DIF was not performed. |
| Holtsche et al. (31) | ELISA was not performed. |
| Horváth et al. (32) | The number of patients positive to DIF was not specified |
| Le Saché-de Peufeilhoux et al. (33) | DIF was not performed. |
| Meijer et al. (34) | Results regarding BP180 and BP230 were not presented separately. |
| Özkesici et al. (35) | The number of patients positive to DIF not specified. |
| Prüßmann et al. (36) | Patients were not affected by blistering diseases. |
| Liu et al. (37) | The number of patients positive to DIF was not specified |
| Ramos et al. (38) | DIF was not performed |
| Ronaghy et al. (39) | DIF was not performed. |
| Shimanovich et al. (40) | ELISA was not performed. |
| Van Beek et al. (41) | DIF was not performed. |
| Wieland et al. (42) | DIF was not performed. |
| Koga et al. (43) | DIF was not performed. |
| Tsutsumi et al. (44) | No information on previous immunosuppressive therapies received by the patient |
| Kumar et al. (45) | The ELISA was done on the patient’s serum concurrently with DIF irrespective of previous treatment. |
