# Supplementary material for: Diagnostic Agreement in Subepidermal Blistering Diseases: Is ELISA Test Reliable as Direct Immunofluorescence? A Systematic Review, Meta‐Analysis, and Trial Sequential Analysis
Source: J Oral Pathol Med. 2025 Dec 12;55(4):391–418. doi: 10.1111/jop.70088 (PMC13065897; doi:10.1111/jop.70088)
Supplement: Supplementary file 2 — Table S2: A summarized scoring systems of diseases severity. [file JOP-55-391-s001.docx]

**E-Table 2.** A summarized scoring systems of diseases severity.

| **Scoring system** | **Target disease** | **Items** | **Score range** | **Notes** |
| --- | --- | --- | --- | --- |
| BPDAI (Bullous Pemphigoid Disease Area Index) | Bullous pemphigoid | Skin lesions (blisters, erosions), urticarial lesions, mucosal involvement, pruritus (VAS) | Max 360 | Developed for BP; separates disease activity from itch |
| PDAI (Pemphigus Disease Area Index) | Pemphigus vulgaris & foliaceus | Skin activity, scalp activity, mucosal lesions and post-inflammatory damage | Max 263 | More sensitive and detailed than ABSIS; gold standard in trials |
| ABSIS (Autoimmune Bullous Skin Disorder Intensity Score) | Pemphigus | BSA (body surface area), mucosal involvement, oral discomfort (VAS) | Max 206 | Combines body surface area with patient symptoms |
| ODSS (Oral Disease Severity Score) | Mucosal involvement (e.g., PV, MMP) | 17 oral sites (0–2 pts each) + oral pain (VAS) + activity score | Max 106 | Sensitive to change in oral lesions |
| MMPDAI (Mucous Membrane Pemphigoid Disease Area Index) | Mucous membrane pemphigoid | Mucosal lesion activity, scarring | variable | Adapted from BPDAI for mucosal disease |
| EB-DASI (Epidermolysis Bullosa Disease Activity and Scarring Index) | Epidermolysis bullosa | Disease activity (wounds, erosions) and damage (scarring, syndactyly) | Max 506 | Complex but comprehensive tool for EB trials |
| Ikeda Score | Pemphigus vulgaris | Extent of lesions, mucosal involvement, Nikolsky phenomenon, number of new blisters per day | Max 12 | Simple, used mainly in Japanese literature |
| DLQI (Dermatology Life Quality Index) | Any dermatological disease | 10 questions on QoL (function, symptoms, emotions) | Max 30 | Generic tool, widely used in dermatology studies |
